# New-Onset Diabetes Mellitus With Exposure to Ledipasvir and Sofosbuvir

**DOI:** 10.1177/2324709615623300

**Published:** 2015-12-29

**Authors:** Resmi Premji, Nira Roopnarinesingh, Nazia Qazi, Eric S Nylen

**Affiliations:** 1 George Washington University Hospital, Washington, DC, USA; 2 VA Medical Center, Washington, DC, USA

**Keywords:** new-onset diabetes mellitus, ledipasvir/sofosbuvir, chronic hepatitis C, glucose intolerance

## Abstract

The combination therapy of ledipasvir/sofosbuvir was approved by the Food and Drug Administration in 2014 for the treatment of chronic hepatitis C. Although hyperglycemia is not well known to occur with its use, we present 2 cases of new-onset diabetes mellitus and a review of the literature suggesting an adverse event association. In the first patient with HIV, we postulate that ledipasvir/sofosbuvir increased the levels of tenofovir and thereby potentiated hyperglycemia. In the second case of a patient with prediabetes, ledipasvir/sofosbuvir appeared to increase insulin resistance. A literature review further supported an association of hyperglycemia and the use of ledipasvir/sofosbuvir. Hence, clinicians should be cautious about worsening of glucose intolerance, and more studies are warranted to explore the underlying mechanism.

## Introduction

The combination therapy of ledipasvir/sofosbuvir (ie, Harvoni) was approved by the Food and Drug Administration in 2014 for eradication of chronic genotype 1 hepatitis C virus (HCV) infections. Clinical trial data have demonstrated this combination to be well tolerated. An adverse glycemic event, as described herein, either independently or in combination is not mentioned with the current drug product information. In this case report, we present 2 patients who had new-onset diabetes mellitus related to the combination therapy of ledipasvir/sofosbuvir.

## Case Report

### Case 1

A 58-year-old nonobese (body mass index [BMI] 24 kg/m^2^) African American male with history of chronic HCV (genotype 1b) and HIV presented to the emergency room with polyuria, polydipsia, and fatigue for 2 weeks duration. Laboratory analysis indicated elevated plasma glucose of 764 mg/dL with an anion gap of 12. He did not have known diabetes mellitus in the past and there was no family history of diabetes. History was significant for recent addition of ledipasvir/sofosbuvir 4 weeks prior to presentation. He was also on tenofovir as a part of highly active antiretroviral therapy. Hemoglobin A1c 1 month before starting ledipasvir/sofosbuvir was 5.5% and currently increased to 10.5%. There was no other ascertainable etiology for sudden new-onset hyperglycemia including use of glucocorticoids, diuretics, statins, atypical antipsychotics, or quinolones. No focus of infection could be identified and serum lipase was normal at 31 U/L. A potential drug-drug interaction between ledipasvir/sofosbuvir and tenofovir was suspected. Hence, the antiretroviral regimen was changed from emtricitabine/rilpivirine/tenofovir (Complera) and raltegravir to rilpivirine/zidovudine and raltegravir. He was continued on ledipasvir/sofosbuvir for a total duration of 12 weeks. Over the next several months, the insulin dose had to be titrated down and eventually stopped due to several bouts of hypoglycemia. Hemoglobin A1c dropped to 6.0% within the next 3 months. At present, the patient is on diet control alone.

### Case 2

The second patient is a 55-year-old obese (BMI 38 kg/m^2^) African American male with chronic HCV (genotype 1b) who presented to the emergency room with polyuria and polydipsia. Plasma glucose was 548 mg/dL with an anion gap of 14. He was started on ledipasvir/sofosbuvir 8 weeks prior to presentation at which time random serum glucose levels had ranged from 70 to 140 mg/dL. Hemoglobin A1c was 6.3% before starting ledipasvir/sofosbuvir and more than 14% on presentation. He was only on diet control and exercise for the management of prediabetes. Similar to the first patient, there was no other clear etiology for the hyperglycemia including history of exposure to glucocorticoids, diuretics, statins, atypical antipsychotics, or quinolones. No ongoing infection was identified and there was no evidence of pancreatitis. He was initially managed with parenteral insulin and was subsequently changed to basal insulin and metformin. Random glucose levels 1 month after discharge ranged from 80 to 140 mg/dL. Over the next 3 months after initiation of hyperglycemia treatment, his BMI improved to 35 kg/m^2^.

## Discussion

The combination use of ledipasvir/sofosbuvir is becoming a very effective treatment modality for HCV with hitherto minor side effects. Ledipasvir is a potent inhibitor of HCV nonstructural protein 5A (NS5A), a viral phosphoprotein that plays an important role in viral replication, assembly, and secretion. Sofosbuvir is an inhibitor of HCV nonstructural protein 5B (NS5B), also a viral phosphoprotein, which leads to cessation of HCV viral RNA replication in all HCV genotypes.^1^ Although hyperglycemia is not a well-known adverse reaction with ledipasvir/sofosbuvir, these 2 cases of sudden new-onset diabetes suggest plausible mechanisms since no other secondary causes of hyperglycemia were identified. Thus, ledipasvir can increase the serum concentration of tenofovir and thereby potentiate one of its known adverse effects, hyperglycemia, as suggested in the first case substantiated with removal of tenofovir.^2^ The serum concentration of tenofovir can increase in the presence of agents such as ritonavir and cobicistat, and concurrent administration is not recommended as per the drug product information of ledipasvir/sofosbuvir. The second patient was obese and had prediabetes on diet control alone at the time of initiation of ledipasvir/sofosbuvir. The hyperglycemia improved significantly after starting metformin and basal insulin. A possible mechanism in this case could be drug-induced triggering of aggravated insulin resistance in view of the salutary weight and metabolic response to metformin treatment.^3^

In support of our findings, a review of the supplementary data in the original phase 3 trial (ie, the ION-3 clinical trial)^4^ showed that in those who received ledipasvir/sofosbuvir for 12 weeks, the hyperglycemia rates (using grades defined by to the Common Terminology Criteria for Adverse Events, hyperglycemia, grade 1: serum glucose > upper limit of normal—160 mg/dL; grade 2: >160-250 mg/dL; grade 3: >250-500 mg/dL: and grade 4: >500 mg/dL)^5^ were 33.3% (grade 1), 11.6% (grade 2), and 2.3% (grade 3). Those who received the medication for 8 weeks had hyperglycemia at the following rates: 21.4% (grade 1), 7.9% (grade 2), and 1.4% (grade 3). Of note, these adverse glycemic changes were not emphasized in the study’s main findings in contrast to adverse effects such as hyperbilirubinemia and anemia albeit with rates similar.

As with prior treatment modalities for hepatitis C,^6^ these 2 cases raise concern regarding adverse effects related to ledipasvir/sofosbuvir. Currently, there are no guidelines on monitoring of hyperglycemia in patients using ledipasvir/sofosbuvir. However, in view of these cases, we suggest clinicians to be vigilant in patients with high-risk features such as obesity, glucose intolerance, and/or strong family history of diabetes. We also recommend observing caution when using ledipasvir/sofosbuvir concurrently with drugs that already increase the risk of dysglycemia.^7^ Monitoring of glucose or hemoglobin A1c before and during treatment could be considered in the aforementioned circumstances.
